# Detection of human Papillomavirus 18 in cervical cancer samples using PCR-ELISA (DIAPOPS)

**Published:** 2011-12

**Authors:** N Raji, M Sadeghizadeh, K N Tafreshi, E Jahanzad

**Affiliations:** 1 Sciences and Research Unit, Islamic Azad University, Tehran, Iran; 2 Department of Genetics, Faculty of Basic sciences, Tarbiat Modares University, Tehran, Iran; 3 Department of Pathology, School of Medicine, Tehran University of Medical Sciences

**Keywords:** DIAPOPS, human papillomavirus, cervical cancer

## Abstract

**Background and Objectives:**

Human Papillomavirus (HPV) infection is a major risk factor for adenocarcinoma of the cervix. The high-risk types of the virus such as HPV16 and HPV18, which possess the E6 and E7 oncogenes, are responsible for approximately 50% of all cervical cancers. A rapid, sensitive and specific test has been proposed for detection of HPV to improve cervical cancer screening programs.

**Objectives:**

The aim of this study was to develop a fast PCR-ELISA assay designated as DIAPOPS (Detection of Immobilized Amplified Products in a One Phase System)for detection of HPV16 and HPV18 types in SCC samples and Pap smears. The type specific primers and probes were designed for PCR and PCR-ELISA. The amplified products were hybridized with a specific *biotin*-labeled probe for HPV18 inner amplicons. The hybrids were detected with *peroxidase conjugated* avidin. The test was performed on the paraffin block and Pap smear samples from the cervical cancer patients, and the results of DIAPOPS were compared with conventional PCR assay.

**Results:**

The 70 samples (SCC and Pap smear samples) were collected from Imam Khomeini and Mirzakoochak Khan Hospitals in Tehran. The PCR-based method detected six HPV16 positive, three HPV18 positive and Two HPV33 positive samples. DIAPOPS results were compared with the conventional PCR results and they showed an increase in sensitivity of the DIAPOPS test. Not only all of them were confirmed by PCR-ELISA but also three samples that conventional PCR showed negative for HPV18, were demonstrated positive by the PCR-ELISA method.

**Conclusion:**

The results of the study show that modified PCR-ELISA assay is more sensitive to detect HPV types and can be used for diagnostic purposes.

## INTRODUCTION

High risk human papillomavirus DNA has been shown to be present in 99.7% of cervical cancers worldwide (1), therefore, high risk HPV testing may have implications for the clinical management of women with cervical lesions (2) and primary screening for cervical cancer.

To date, the detection of HPV genotypes has been done predominantly by L1 general or consensus primer PCR assay (3) and using the commercially available liquid hybridization assay, Hybrid Capture 2 (2). The general primer PCR assays enable the detection of a broad spectrum of mucosotropic HPV types. Since the primers anneal to a highly homologous region of the HPV types, this allows specific HPV typing. Among all of the general primer PCR assays, the GP5+/GP6+ and MY09/MY11 PCR systems are most frequently used and clinically evaluated (4). Following the general primer PCR assays, the HPV type determination has been done by nucleotide sequencing (5) or oligonucleotid probe hybridization (Southern dot blotting) and EIA (6) of the PCR products. However, all of these methods are very laborious.

Hybridization with type-specific probes using PCR-ELISA is the alternative method that has been used to identify HPV genotypes following amplification with general and consensus primers (7). Here we developed an easy and rapid PCR-ELISA (DIAPOPS) assay that is suitable for clinical purposes.

## MATERIALS AND METHODS

**Clinical Samples.** A total of 70 cervical specimens were collected from the women attending general practitioner clinics during routine cytological screening at the Department of pathology of the Imam Khomeini and Mirza Kochak Khan hospitals. Sixty four samples were paraffin-embedded tissue that distinguished as a sqamus cell carcinoma (scc) using cytological tests. Six fresh Pap smear samples were collected from the cervical cancer patients and suspended in 200 ml of physiological serum.

**Sample preparation.** For detection of HPV in paraffin-embedded specimens, the 5-µm sections were cut and deparaffinized *with* xylene. The Lysis mixture (200 µl) was added to the samples and incubated overnight at 37°C. Then 100µl of phenol-chloroform-isoamilalchol (25:24:1) was added. The solution was thoroughly mixed and centrifuged, and the top phase was extracted. DNA was precipitated by the addition of 1ml of 95% ethanol and 50µl of 3.5 M sodium acetate (pH 3.5). Following the centrifugation at 13000 rpm, the pellet was washed twice with 70% ethanol, dried in the room, and re suspended in 30µl of deionized water. 17 samples were also extracted using the DNA extraction kit from bioneer Inc. (Soath Korea)

**Positive controls.**The DNA was extracted from the Hela cell line (Pasteur Institute, Iran) using DNA extraction kit from bioneer Inc. The extracted DNA was used as a positive control. In order to test the cross reactivity between our system and other viruses, the system was checked with some other genome of viruses including: Adenovirus, Hepatitis B virus (HBV) and Herpes simplex virus (HSV).

**Primers and Probes.**The primers were designed and synthesized for the sequences of the E1 genes of HPV16, HPV18, HPV31 and HPV33 in the intermediate group as described by Kanazawa and et al. (8)**. The HPVs amplified products were accurately identified by their product size. In order to detect the DNAs of HPV 16, 18, 31 and 33, four different PCR reactions were performed for each of samples. The primers for the PCR-ELISA were designed by adding a 10 thymidine residues and a phosphate group to the 5' end of the forward primers. The designed probe was biotinylated at the 5’ end and annealed to the region between forward and reverse primers in the reverse strand. The sequence of the probe was checked for hairpin loop and dimer formation for hybridization with coated primer. All PCR primers, and PCR-ELISA primers and probe were supplied by Bioneer (South Korea). The primer and the probe sequences are illustrated in Table 1.

**Table 1:** Primer and probe sequences.

Primers	Sequences (5’→3’)	Tm°C	Amplified DNA size (bp)
HPV-16,F	ACCCAGTATAGCTGACAGT	68.3	512
HPV-16,R	CTCGTTTATAATGTCTACACA	68.3	
HPV-31,F	ATAGCAATTTTGATTTGTC	68.7	188
HPV-31,R	AAACTCATTCCAAAATATG	68.7	
HPV-18,F	ATAGCAATTTTGATTTGTC	64.3	415
HPV-18,R	AAACTCATTCCAAAATATG	64.3	
HPV-33,F	ATGCACAACTTGCAGATTC	64.6	334
HPV-33,R	AAACTCATTCCAAAATATG	64.2	
DIAPOPS forward primer HPV-18	P-TTTTTTTTTT- ATAGCAATTTTGATTTGTC		
DIAPOPS probe HPV-18	biotin-TGTGCAAACATTATAGGCGAGCC		

**PCR Amplification.** The PCR was performed in 25 µl reaction mixtures, using the Amplisense taq DNA polymerase (CinnaGen, Tehran, Iran). Thermal cycling of amplification mixture was performed in the gradient PCR for a total of 35-40 cycles. The cycle represents denaturing for 1 minute at 94°C, annealing for 1 min at 49°C for HPV16 and 31 and 44°C for HPV18 and 33, and a primer extension for 1 min at 72°C.

The PCR Products were detected on 1% (w/v) agarose gel electrophoresis. The amplified fragments were verified using a Gene Ruler DNA Lithnavia ladder (Fermentas Inc.).

The PCR-ELISA positive samples that could not be detected on agarose gel were tested using a 6% (w/v) polyacrylamide gel electrophoresis.

**PCR-ELISA (DIAPOPS).** PCR-ELISA was perform-ed according to the Nunc protocol with some modifications. The 5′-monophosphatated, ten thymidilated forward primers were coated on the NucleoLink strips (Nunc Denmark) that is suitable for covalent binding of oligonucleotides. The coating was performed using a fresh coating buffer (forward primer 100 nM, cardiaamide 10 mM, imidazole 10 mM; pH = 7) for 24 h at 50°C, and 100 µl for each well. Following the 3 times washing steps, each time for 5 minutes with washing buffer (Trist-HCl 100 mM, NaCl 150 mM, tween 20 0.1%; pH = 7.5) and deionized water, the PCR reaction was performed in each well.

The PCR condition was at the same as PCR described above, except that the reaction was done in the coated NucleoLink stripe and in the reaction mixture, the forward primer (coated primer) was 1/8 the reverse primer, and 2.5 µl BSA 10% were used in the 25 µl reaction mixture.

In order to detect the HPV DNA types, the PCR products were treated by the denaturing buffer (NaOH 0.2 M, tween 20 0.1%), and then were washed with the washing buffer (Trist-HCl 100 mM, NaCl 150 mM, tween 20 0.1%; pH = 7.5). At the next step, the hybridization buffer (biotinylated probe 50 nM, SSC 5X{from the SSC 20X containing, NaCl 175.3 gr, Sodium citrate 88.2 gr, adjust with water to 1 lit; pH = 7} tween 20 0.1%, BSA 0.5%) was added to each well. The wells were sealed and incubated for just 1 h rather than 20 h in 50°C. Then, the wells were washed with buffer containing SSC 0.5X and tween 20 0.1%, five times and each time for 5 minutes at the room temperature, except the third round that was done in the 50°C and for 15 minutes. The detection buffer (1/5000 diluted HRP-avidin, Tris-HCl 100 mM pH = 7.5, NaCl 150 mM, tween 20 0.1%; BSA 0.5%) was added to each well, and the wells were sealed and incubated in 50°C for 30 minutes. After three times washing the wells with the washing buffer, 100 µl of ready to use TMB mixture were added to each well. The reaction of the enzyme was blocked by 0.1 M H2SO4, after appearance the color in positive controls. The results were read on the ELISA reader.

**Cut-off value of probability.** In order to determine cut-off value of probability for DIAPOPS, 64 negative samples were used and after the detection procedure, the results were read by the same ELISA-reader that used or clinical samples. The positive cut-off value for PCR-ELISA was determined according to the equation below for probability of 100%: positive cut-off = X +Z _(α/2)_. σ/√N, where the Z _(α/2)_ is equal 3, Average (X) = 0. 308174, standard deviation (σ) = 0. 174754 and N = 64, positive cut-off is as calculated below: Cut-off. = 0. 308174 + 3 (0. 174754/√64) = 0.373. A test was supposed positive, if optical density of the sample was higher than the cut-off value (9).

## RESULTS

**Fig. 1:**
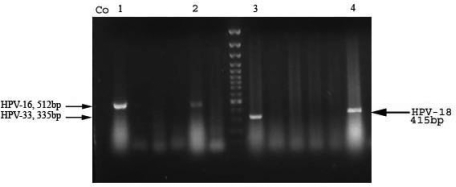
The figure shows the results of conventional PCR. CO, negative control, lines 1 and 2: HPV16, line3: HPV33, and line 4:HPV18.

**PCR based detection of HPV types.** In this study, 70 clinical samples were tested with PCR for detection the HPV types 16, 18, 31 and 33. The results of the PCR products gel electrophoresis were shown in Fig. 1. Six samples were detected as HPV16 Positive, three samples as HPV18 positive and two samples as HPV33 positive from total of 70 samples.

**DIAPOPS.** The PCR-ELISA assay was designed for HPV18 and the results of the test was compared with the conventional PCR method. Following determination of positive cut-off, all the samples that had the OD above the positive cut-off, were supposed as infected samples. Sixty four negative samples were used for cut-off in DIAPOPS and then all samples were tested for detection of HPV18 using PCR-ELISA. The results showed that DIAPOPS not only detected all the samples which were reported as positive in conventional PCR, but also it detected three samples as a positive HPV18 that were reported as negative HPV18 samples in the PCR.

In order to confirm the results, the PCR products of DIPOPS positive samples were tested on 6% (w/v) polyacrylamide gel electrophoresis and the results of PCR-ELISA were confirmed on the PAGE. The comparison of three methods is shown in Table 2.

**Table 2:** The results of conventional PCR, DIAPOPS and PAGE electrophoresis for the detection of HPV18.

Samples	B1	H3	A4	E6	H7	A5
Agarose results	+	+	+	–	–	–
Polyacrylamide result	+	+	+	+	+	–
DIAPOPS result	+	+	+	+	+	+
conclusion	+++	+++	+++	–++	–++	––+

## DISCUSSION

Cervical cancer is one of the main risk factors in mortality of women worldwide. Evidence shows strong relation between cervical cancer and HPV infection. HPV is not able to be cultured in the laboratory from clinical samples and immunologic assays are not adequate for detection of HPV infections (10). The primary detection tools have been cytology and histology. The sensitivity of cytological method that uses Pap smear samples is too low (11).

The alternative HPV testing is based on detection of HPV DNA in cervical specimen that is performed using PCR technique. Conventional PCR has higher sensitivity and efficiency of detection when compared to histological examination. The majority of studies using PCR have used consensus primers to amplify HPV types in a single PCR amplification. These primers target conserved regions of HPV genome such as the L1 capsid gene. The MY09-MY11 primers target a 450-bp fragment within the HPV L1-ORF (10). The type specific PCR assays are based on the sequence variations present in the E6 and E7 genes of HPV subtypes (8).

The advantages of detecting viral DNA by PCR include rapidity, sensitivity, ability to detect nonviable virus and potential elimination of contaminating microorganisms. Traditionally, PCR products are detected by agarose gel electrophoresis. This detection system has several disadvantages. It is time consuming and difficult to standardize since the results are not based on figures. Gel electrophoresis gives a poor detection limit, is not specific and only subjective product quantification is possible unless a gel scanner is available. These problems can be overcome by PCR-ELISA.

Venturoli and Zerbini in 1998 used PCR-ELISA method for detection of HPV. They used consensus primer pair MY09-MY11 in PCR amplification. The amplified DNA was labeled with digoxigenin and the hybridization occurred between PCR product and biotinylated probe. The plat wells coated with streptavidin, therefore hybrid entraps by interaction between biotin and streptavidin. The detection is performed by Dig antibody conjugated enzyme (7, 12). In this method, amplification system and the detection system are separate; therefore, there is risk of contamination. Another disadvantage of this method is that the detection system by DIG and anti-Dig is expensive. In this study, DIAPOPS method was used and these problems can be overcome using the Nucleolink strips. Since there is no transfer of amplified products between amplification system and the detection system, the risk of contamination carry over is greatly reduced. As well as the high sensitivity and specificity of this DIAPOPS, there is also the advantage that with this procedure the PCR product is rapid, easy and objective it requires no electrophoresis, UV light, or darkroom and furthermore the use of toxic chemical agents such as ethidium bromide is avoided (9). Moreover, the technique allows the simultaneous handling of a large number of samples and can be automated.

In this study, the HPV type16, 18, 33 and 31 were detected by PCR in suspected cervical cancer samples and DIAPOPS method was designed for HPV18. Results were compared with conventional PCR. Seventy samples (SCC and Pap smears samples) were collected from Imam Khomeini and Mirzakoochak Khan Hospitals in Tehran. Of the70 specimens analyzed by PCR, 11 were HPV DNA positive (15.71%), six samples were detected as HPV16 positive (8.5%), three samples as HPV18 positive (4.3%) and two samples as HPV33 positive (2.85%). From a total of 70 samples for HPV18 in PCR-ELISA, 6 samples were detected as positive (8.5%); therefore, positive samples increased from 4.3% by gel electrophoresis to 8.5% by PCR-ELISA. These results are the same as other studies. Zerbini and Venturoli reported that of the 176 cytological specimens, 106 were HPV DNA positive (60.2%); of the 106 HPV DNA positive specimens, 22 were positive by agarose gel electrophoresis (20.7%) whereas 84 (79.2%) HPV positive specimens could be typed by the PCR-ELISA (12).

The results of the study show that PCR-ELISA method modified in this study was more sensitive to HPV type detection in comparison to PCR alone. This method can be used for HPV oncogenic viral type detection in one day for clinical purposes.
